# Optimal surveillance strategy for invasive species management when surveys stop after detection

**DOI:** 10.1002/ece3.1056

**Published:** 2014-04-11

**Authors:** Gurutzeta Guillera-Arroita, Cindy E Hauser, Michael A McCarthy

**Affiliations:** School of Botany, The University of MelbourneParkville, 3010, Victoria, Australia

**Keywords:** Cost-effectiveness, detectability, imperfect detection, optimization, removal design, survey design

## Abstract

Invasive species are a cause for concern in natural and economic systems and require both monitoring and management. There is a trade-off between the amount of resources spent on surveying for the species and conducting early management of occupied sites, and the resources that are ultimately spent in delayed management at sites where the species was present but undetected. Previous work addressed this optimal resource allocation problem assuming that surveys continue despite detection until the initially planned survey effort is consumed. However, a more realistic scenario is often that surveys stop after detection (i.e., follow a “removal” sampling design) and then management begins. Such an approach will indicate a different optimal survey design and can be expected to be more efficient. We analyze this case and compare the expected efficiency of invasive species management programs under both survey methods. We also evaluate the impact of mis-specifying the type of sampling approach during the program design phase. We derive analytical expressions that optimize resource allocation between monitoring and management in surveillance programs when surveys stop after detection. We do this under a scenario of unconstrained resources and scenarios where survey budget is constrained. The efficiency of surveillance programs is greater if a “removal survey” design is used, with larger gains obtained when savings from early detection are high, occupancy is high, and survey costs are not much lower than early management costs at a site. Designing a surveillance program disregarding that surveys stop after detection can result in an efficiency loss. Our results help guide the design of future surveillance programs for invasive species. Addressing program design within a decision-theoretic framework can lead to a better use of available resources. We show how species prevalence, its detectability, and the benefits derived from early detection can be considered.

## Introduction

The number of introductions of non-native species is increasing worldwide due partly to the intensification of trade, transport, and travel (Meyerson and Mooney 2007). Introduced invasive species are a major driver of biodiversity loss, as they compete with or prey upon native species, often dramatically altering natural habitats (Millennium Ecosystem Assessment 2005). The Convention on Biological Diversity (CBD) identifies the impacts of invasive species on biodiversity as a critical global issue, and in Article 8(h), it obliges parties to *“prevent the introduction of, control or eradicate those alien species which threaten ecosystems, habitats and species.”* Invasive species are also a problem for economic activities, such as agricultural production, where they can lead to large economic losses (Sinden et al. 2004; Pimentel et al. 2005). For instance, Williams et al. (2010) estimated the annual costs of invasive non-native species to Great Britain at £1.7 billion. The cost of controlling an invasion increases substantially as the area occupied grows (Tobin et al. 2014), and managing a species during the early stages of invasion can often save resources in the long term (Wittenberg and W Cock 2001; Williams et al. 2010).

Identifying the presence of an invasive species at a site early in the invasion process requires monitoring. As species detection is usually imperfect (Yoccoz et al. 2001), the presence of the species might be overlooked during surveys. Imperfect detection is not only a problem for mobile species, but is also an issue for sessile species such as plants (Garrard et al. 2008; Moore et al. 2011; Chen et al. 2013). Declaring an invasive species absent from a site where it is present can ultimately lead to increased management costs (unless the population goes extinct by itself). On the other hand, the chance of overlooking species presence is reduced as the effort invested in monitoring is increased. Therefore, a trade-off between monitoring and management costs exists, and a question arises of how much one should invest in invasive species surveillance. Rather than setting the amount of survey effort at an arbitrary level, this problem can be framed in terms of cost optimization following a decision-theoretic framework. Past studies have determined the optimal survey investment for detecting new incursions (Mehta et al. 2007), managing an incursion (Homans and Horie 2011; Epanchin-Niell et al. 2012), declaring eradication (Regan et al. 2006; Rout et al. 2011), and managing multiple incursion stages (Moore et al. 2010; Rout et al. 2011).

Hauser and McCarthy (2009) developed a survey design for invasive species management via a cost optimization that balances monitoring and management costs in different locations. This approach determines the optimal level of surveillance at a given point in time and proved useful for conducting surveys to detect the invasive plant orange hawkweed (*Pilosella aurantiaca*), which occurs at low densities in southeastern Australia (Curran 2013; Herbert et al. 2013). This work found that intensive surveillance is beneficial at sites that have high probability of species occurrence and when savings associated with early detection are large. The optimal investment of survey effort is a nonlinear function of these factors.

In their optimization, Hauser and McCarthy (2009) assumed for simplicity that the cost of surveying a site was the same regardless of whether the species was detected or not. However, in the surveillance of invasive species, we can expect that surveys would normally be terminated once a species is detected, as species detection is all that is needed for triggering management action. Once a species is detected at a site, remaining resources can be directed to management or to the monitoring of other sites. Hence, terminating surveys after detection will lead to a more efficient invasive management program. We expect that considering this characteristic of the sampling will mean that the optimal level of survey effort at each site will differ from the solution of Hauser and McCarthy (2009), especially when the species is encountered frequently.

In this paper, we revisit the problem of optimal surveillance in invasive management programs, with an explicit consideration of surveys ending at any site when the species is detected. Our aim is threefold: (1) to provide the analytical solution to the stated cost optimization problem, as well as code for its implementation, as a tool for designing future surveys; (2) to evaluate how the expected efficiency of an invasive management program compares under both monitoring strategies (i.e., continuing or stopping after detection); and (3) to assess the impact of disregarding that surveys stop after detection during survey design. We consider the two scenarios contemplated by Hauser and McCarthy (2009): one without budgetary constraints and another in which the resources that can be spent for surveillance are constrained.

## Methods

### Scenario considered

We consider the following scenario, as proposed by Hauser and McCarthy (2009). A site is surveyed, and if the species is detected, then the site is managed for its eradication. If the species is not detected, the site is not managed under the assumption that the species is not present. However, the species could have been present and missed, with the lack of early management leading to a wider infestation that ultimately requires higher management costs. Unlike Hauser and McCarthy (2009), here, we assume that surveys at a site are immediately discontinued after first detection and that the unused survey effort can be utilized elsewhere. In the occupancy estimation literature, a design in which surveys end after first detection is referred to as a “removal” design, given that sites are removed from the set of sites being sampled after the species is detected (MacKenzie and Royle 2005; MacKenzie et al. 2006 p. 89). Hereafter, we adopt this terminology to refer to this type of sampling approach, and in contrast, we use the term “nonremoval” to refer to surveys that do not stop after detection.

### Detection model

We modeled the detection of the species at occupied sites as a Poisson point process with rate *γ*_*i*_ (Cox and Isham 1980), where *γ*_*i*_ is the mean number of detections per unit of survey. Under such a model, the time to first detection is exponentially distributed and the probability to detect the species (at least once) at an occupied site in a survey of duration *L*_*i*_ is 1 − exp (−*γ*_*i*_*L*_*i*_). Following a “removal” design, the site is surveyed until the species is detected or until a maximum survey length, 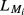, is reached, whatever comes first. In Appendix S1, we show that the expected length of surveys at occupied sites is


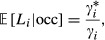


where 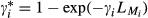 is the probability of detecting the species at an occupied site before the survey terminates. Considering that the species might be absent from the site, the unconditional expected survey length is


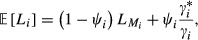
(1)

where *ψ*_*i*_ is the probability that the site *i* is occupied by the species. It obviously holds that 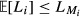 (i.e., survey duration cannot exceed the maximum allowed 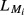) and that 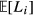 approaches 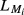 as *γ*_*i*_ or *ψ*_*i*_ approaches zero (i.e., the expected survey length is greater at sites unlikely to contain the species, or if the species is very hard to detect).

### Optimization

We assume that our study covers *n* sites and we want to determine the optimal amount of survey effort to be spent at each of these sites so that we minimize overall costs, including both those from surveillance and management. That is, we are seeking the optimal values of 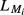. The expected total cost at site *i* is


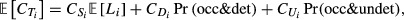
(2)

where Pr(occ&det) = *ψ*_*i*_ 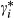 is the probability that the site is occupied and the species is detected, Pr(occ&undet) = *ψ*_*i*_ (1−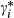) is the probability that the site is occupied and the species is not detected, 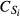 is the survey cost per unit of survey length, 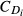 is the cost of early management, which is incurred when the species is detected, and 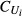 is the cost of late management, which is ultimately incurred if the species is present but not detected during the surveys. The above formulation can apply to the case where the invasive species has a non-negligible intrinsic probability of going extinct at a site left unmanaged (*ε*), by replacing 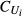 with 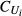
(1 − *ε*), thus reflecting the *expected* costs of late management.

Without loss of generality, we hereafter express costs in terms of costs of a survey of unit length, that is, 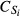 = 1. We can express equation 2 as


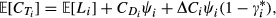
(3)

where *ΔC*_*i*_ = 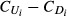 denotes the increment in cost derived from not managing the site in time before widespread infestation. We assume that, as could be expected, 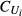 is considerably larger than 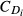 (i.e., *ΔC*_*i*_ > 0), and hence, early management is beneficial at sites where the species is present. Equation 3 shows the trade-off in costs: as survey effort increases (i.e., 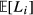 increases), the probability of detecting the species increases (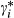 increases), and hence, the expected management costs, which are a function of 1 − 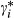, decrease.

In the case where there are no budgetary constraints, the optimal amount of (maximum) survey effort at site *i*, 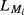, is that which minimizes the expected total cost at the site. Hence, for such a scenario, we obtained the optimal 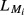 by differentiating the expression in equation 3 with respect to 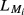, equating to zero and solving (Appendix S2). We denote B* the total expected survey costs over the *n* sites in this unconstrained scenario, that is, 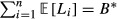.

When there is an overall budgetary constraint for the surveys, the optimization cannot be performed on a site-by-site basis as above. Instead, we ensure that the overall expected survey costs are within the allowed budget. Hence, we formulate the problem as the following constrained optimization


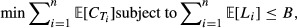


that is, we identify the set of 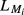 that minimizes the total expected costs summed across all sites, 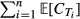, while ensuring that the overall expected survey cost, 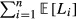, does not exceed the allowed budget *B*. We solved the optimization using the Kuhn-Tucker conditions (Winston 2004, Pp. 670–675; Appendix S3). Note that, if *B* ≥ B*, the budget is effectively not constrained, and the optimal solution corresponds to that of the unconstrained problem.

### Efficiency comparison between “removal” and “nonremoval” sampling methods

We first explored the scenario without budgetary constraints. We computed the optimal allocation of survey effort for a “removal” and a “nonremoval” design for different values of site occupancy probability *ψ*_*i*_ (from 0.1 to 0.9), detection rate at occupied sites *γ*_*i*_ (from 0.1 to 2), three cases of difference in costs between late and early detection (*ΔC*_*i*_ = 10, 20, 50), and two values of cost of early detection (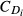 = 2, 10). We evaluated the efficiency of the “removal” design with respect to the “nonremoval” design by computing the ratio between the resulting overall expected costs of the program under the two sampling schemes. Ratios smaller than one indicate that the “removal” design results in lower overall expected costs. We also evaluated the impact of wrongly assuming during survey design that surveys continue after detection (i.e., assume a “nonremoval” design), when in reality a “removal” design is used.

We then compared the efficiency of the “removal” design with respect to the “nonremoval” design under scenarios of constrained survey budget *B*. To add some variability among sites, we generated a hypothetical landscape with 500 sites and assigned to each of the sites a probability of occupancy and a detection rate. We chose these values by drawing from uniform distributions. We explored two occupancy scenarios (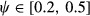 and 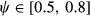) and two detection scenarios (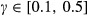 and 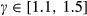). We assumed that the management costs were equal for all sites with 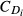 = 2 and *ΔC*_*i*_ = 50. We explored a range of survey budget levels, starting with *B* = 0 and increasing the budget until it was larger than the survey costs corresponding to the optimal level of surveillance under unconstrained conditions (i.e., *B* > B*).

Finally, we assessed the effect that a scaling in the probabilities of occurrence has in determining the optimal allocation of effort. We assumed a scenario with 100 sites with different occupancy probabilities, 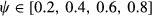, 25 sites of each type. We obtained the optimal level of surveillance for this case and also after scaling the occupancy probability by 0.6. In both cases, we set *γ* = 1 and *ΔC* = 10 for all sites. We swept across survey budget levels, from *B* = 0 to *B* = 300, which ensures survey budget levels larger than the optimal one under no constraints.

## Results

### Optimization results

Assuming no budgetary limitations, the maximum survey length 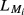 that minimizes the total expected cost at the site is (Appendix S2)


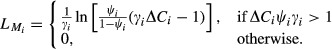
(4)

The optimal level of survey effort increases with increases in the odds of site occupancy (i.e., large 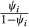) and with increases in the savings from early detection of the species (i.e., large *ΔC*_*i*_). The optimal level of survey effort increases with *γ*_*i*_ when *γ*_*i*_ is not much larger than *ψ*_*i*_*ΔC*_*i*_ but otherwise decreases with *γ*_*i*_.

The solution of the constrained optimization (Appendix S3) indicates that, when *B* < B*, a good design starts by arranging sites in descending order of *ΔC*_*i*_*ψ*_*i*_*γ*_*i*_. Only the top sites are allocated some survey effort. Assuming that the indexing now refers to such ordering, which sites are allocated survey effort is summarized by the following condition


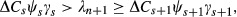


where *s* denotes the number of sites that receive survey effort and *λ*_*n*+1_ takes a value that fulfills the expression in Table 1 and hence depends on the allowed budget *B*, as well as on the values of *ψ*_*i*_, *ΔC*_*i*_, and *γ*_*i*_ at the different sites. The optimal survey length 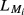 is


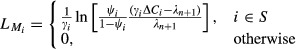
(5)

**Table 1:** Comparison of the analytical optimization results for the cases where surveys stop after first detection (“removal” design) and surveys do not stop after first detection (“nonremoval” design; Hauser and McCarthy 2009)

	Surveys stop after detection (“removal”)	Surveys do not stop after detection (“nonremoval”)
No budget constraint	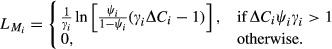	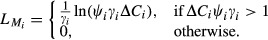
Survey costs constrained *B* < B*	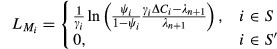	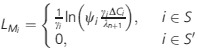
	where *λ*_*n*+1_ fulfills	where
	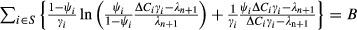 with 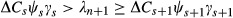	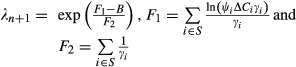 with 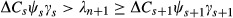
	Note: 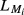 = *f* (*γ*_*i*_, *ψ*_*i*_, *ΔC*_*i*_)	Note: 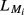 = *f* (*γ*_*i*_, *ψ*_*i*_*ΔC*_*i*_)

where *S* denotes the set of sites that receive survey effort (i.e., sites 1… *s* after being ordered). To facilitate application to real data, we provide code written in R (R Development Core Team 2011) that implements these results (equation 4 and 5), as well as those provided by Hauser and McCarthy (2009), shown in Table S1 (see supplementary material).

### Comparison between sampling methods

Table 1 compares the analytical results that we obtained for the determination of optimal surveillance level considering a “removal” sampling design to the expressions derived in Hauser and McCarthy (2009) assuming a “nonremoval” sampling design. Their expressions are broadly similar to ours, and only the top sites in terms of *ΔC*_*i*_*ψ*_*i*_*γ*_*i*_ are allocated survey effort. However, the determination of how many sites receive survey effort, and the amount of survey effort required at each site before establishing species absence (i.e., 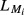) is different. While for the “nonremoval” sampling, the optimal level of surveillance only depends on *γ*_*i*_ and the product *ψ*_*i*_*ΔC*_*i*_ (Hauser and McCarthy 2009), under a “removal” design, it depends on *ψ*_*i*_, *γ*_*i*_ and *ΔC*_*i*_ separately.

**Figure 1:**
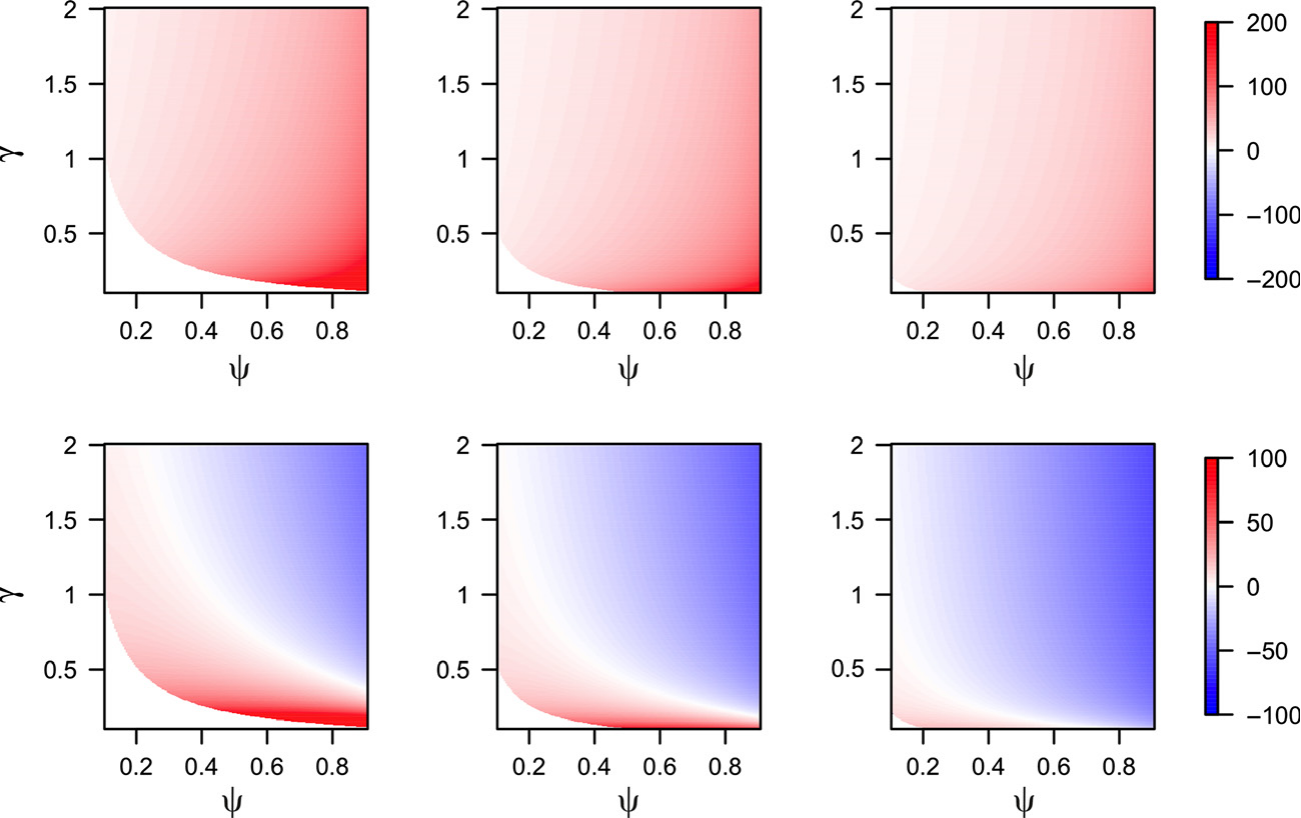
Difference (%) in survey effort under the optimal surveillance strategy between a program based on “removal” surveys and one with “nonremoval” surveys for varying levels of occupancy *ψ*_*i*_ and detection rate *γ*_*i*_, and three scenarios of increased management costs due to late detection *ΔC*_*i*_ = 10, 20, and 50 (columns from left to right). In the first row, we compare the optimal survey length (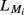) under both schemes. In the second row, we compare the expected survey length that will need to be carried out (i.e., 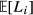 in a “removal” design and 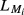 in a “nonremoval” design). Note the difference in scale between the two rows. These plots are independent of the particular values 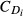 and 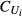.

Looking at the unconstrained solution, it can be seen that 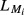 is always larger under the “removal” design (see Appendix S4 for an analytical demonstration). In proportion, the increase in 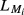 is greater for sites with high *ψ*_*i*_, low *γ*_*i*_, and low *ΔC*_*i*_ (Fig. 1, top row). The increase in 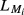 does not necessarily imply that the actual amount of survey effort spent is always larger, given that surveys stop after detection. In fact, the expected amount of survey effort spent with a “removal” design (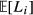) is smaller than the amount of survey effort in a “nonremoval” design when *ψ*_*i*_ and *γ*_*i*_ are large, with the reduction being more generalized as *ΔC*_*i*_ increases (Fig. 1, bottom row).

**Figure 2:**
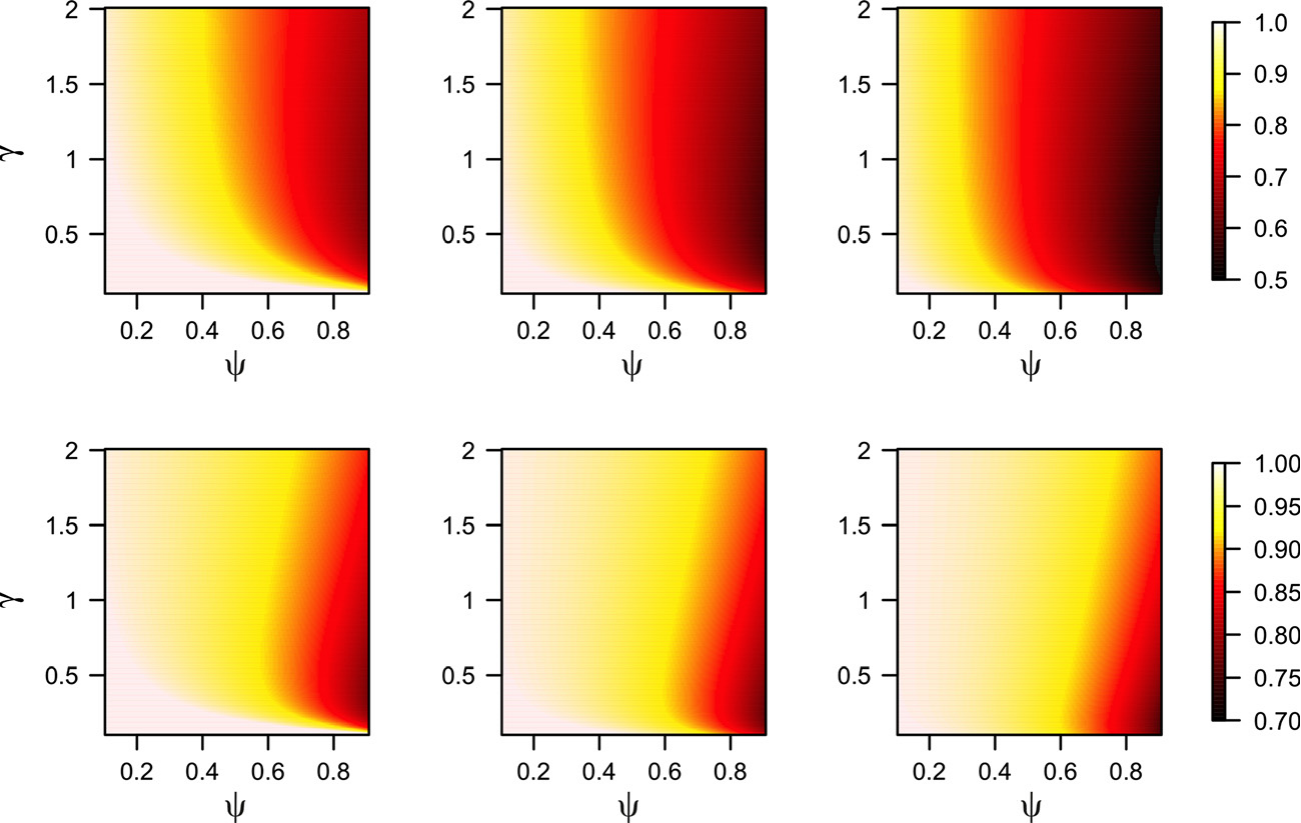
Efficiency measured as the ratio of total expected costs (surveillance plus management) for varying levels of occupancy *ψ* and detection rate *γ*, three scenarios of increased management costs due to late detection *ΔC_i_* = 10, 20, and 50 (columns from left to right) and a cost associated with detection of *C*_*Di*_ = 2. In the first row, we compare a program that uses a “removal” sampling design with a program based on a “nonremoval” design. In the second row, we consider the application of a “nonremoval” design to a “removal” management scenario and we evaluate the loss in efficiency due to terminating nondetection surveys early. Note the difference in scale between the two rows.

The “removal” design improves efficiency, especially when the probability of occupancy is high (Fig. 2, top row), with the results being less sensitive to changes in detection rate. The efficiency gain is more evident as *ΔC*_*i*_ increases, with the ratio of total costs getting as low as 0.5 in the scenarios we explored. As could be expected, the difference in performance was less apparent when 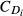 increased (Fig. in Appendix S5) as this means that survey costs matter less. Assuming in the optimization that surveys continue after detection when surveys in practice stop after detection can increase costs (Fig. 2, bottom row). These costs arise from failed detections under the lesser survey effort prescribed by the “nonremoval” optimization.

**Figure 3:**
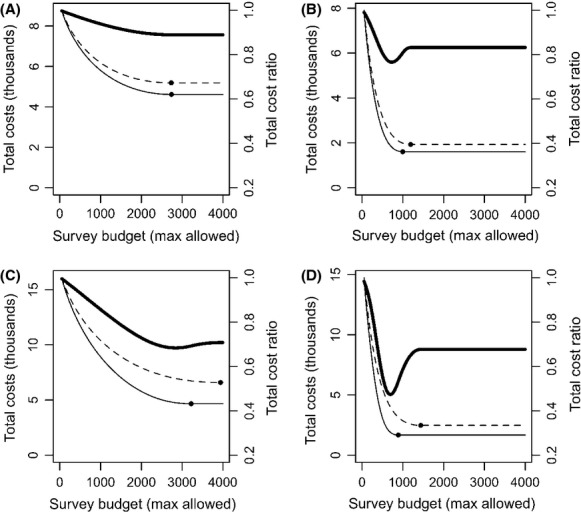
Expected total costs and their ratio as a function of maximum allowed survey budget *B*. Solid line represents the “removal” design, dashed line represents the “nonremoval” design, and the thick solid line is the cost ratio. The dot on the lines represents the optimal amount of survey effort; from this point on, the survey budget is effectively unconstrained. Scenarios: occupancy probability 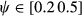 in (A) and (B), 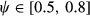 in (C) and (D); detection rate 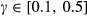 in (A) and (C), 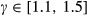 in (B) and (D); 500 sites; *C*_*D*_ = 2; *ΔC* = 50.

**Figure 4:**
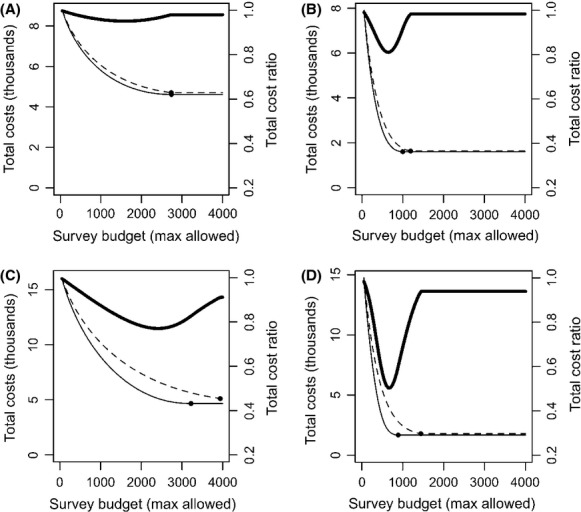
As Figure 3, but comparing the costs of a program based on a “removal” design when the fact that surveys end after detection is either considered (solid line) or disregarded (dashed line) during survey design.

The difference in efficiency between survey methods changes if the survey budget is constrained (Fig. 3). As the survey budget *B* decreases, with either method, the total costs increase toward the value expected when there is no monitoring, that is, 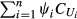. In our scenarios, this was 52(0.2 + 0.5)/2 = 9100 for the cases displayed in Fig. 3A, C and 52(0.5 + 0.8)/2 = 16900 for the cases displayed in Fig. 3B, D. While *B* is small, the difference in the total costs achieved by each of the methods is small, as management costs dominate the expenditure. Hence, the cost ratio is close to one. As *B* increases, the total costs decrease until they reach the costs achieved by the unconstrained optimal survey effort allocation. In some scenarios (e.g.*,* Fig. 3A), the cost ratio between the “removal” and “nonremoval” methods is smallest when the budget is unconstrained. In others (e.g., Fig. 3B–D), the difference in efficiency is more prominent when the survey budget is constrained. For instance, in Fig. 3D, the total costs when “removal” surveys are used is 30% lower than when “nonremoval” surveys are used if the survey budget is unconstrained, but more than 50% lower for particular constrained budget levels. The impact of disregarding that surveys stop after detection can also be greater under constrained scenarios (Fig. 4).

**Figure 5:**
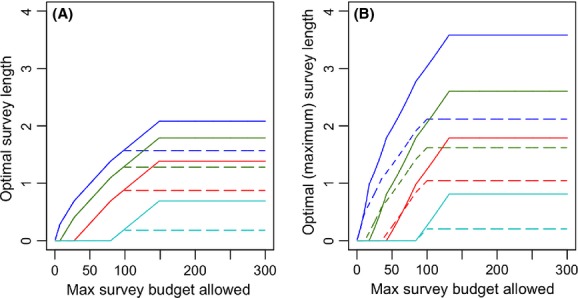
Effect of occupancy scaling on the optimal level of surveillance (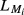) under a “nonremoval” design (left) and a “removal” design (right). In this example, there are 100 sites with four occupancy values, 25 sites of each. Solid lines correspond from bottom to top to *ψ* = 0.2 (cyan), *ψ* = 0.4 (red), *ψ* = 0.6, (green), and *ψ* = 0.8 (blue). Dashed lines correspond to a scenario in which occupancy probabilities are scaled by 0.6. For all sites, *γ* = 1 and *ΔC* = 10. Only under a “nonremoval” design with an effectively constrained survey budget, the optimal level of surveillance is the same regardless of the scaling in occupancy.

A scaling in the probability of occupancy changes the optimal surveillance allocation for unconstrained budgets, as well as for constrained budgets under the “removal” survey design (Fig. 5). For the “nonremoval” design, the solution is the same for both cases, while the budget is effectively constrained, but note that the optimal unconstrained budget level (B*) changes when the occupancy probabilities are scaled.

## Discussion

Spatial prioritization efforts for single species should be concerned not only with the probability of species occurrence, but also with the ability of detecting the species, associated survey costs, and the benefits derived from species detection (Hauser and McCarthy 2009). Our cost optimization approach considers all of these components to guide the efficient allocation of surveillance effort in invasive management programs, with the novelty that here we adopt a realistic sampling scenario in which surveys stop after species detection (i.e., “removal” sampling). We consider scenarios where the survey budget is unconstrained as well as constrained. We note that the latter leads to increased overall costs, as reducing survey effort below the optimal surveillance level implies that the potential benefits of early detection are not fully realized.

Dealing with invasions is difficult. There are different ways to approach the problem of detection and eradication, and, in fact, different invasion problems will have different characteristics. Here, we focus on a single species that is known to have arrived and is identified as damaging. The work we present, as well as that in the original motivating paper by Hauser and McCarthy (2009), provides managers with a relatively simple tool to determine survey effort at the beginning of a management season/campaign. By determining the best allocation of resources at a given point of time, these methods do not explicitly account for the dynamic nature of the invasion. In practical terms, this limitation has the advantage of simplicity for implementation by managers. It has proven useful for seasonal programs such as Victoria's hawkweed eradication effort, where a budget is set and resources are prioritized in spring and a survey is carried out in summer. Between seasons, managers have the opportunity to update occupancy models with the new survey data, predict possible invasion spread, and re-prioritize effort for the next survey.

“Removal” sampling is an appropriate assumption in our invasive species surveillance setting as the focus is only on the detection of the species. Once the species is detected at a site, the decision for early management is triggered. Surveying the site for a longer period of time after first detection brings no direct benefit in terms of the decision-making process. The resources “freed up” once the species is detected are available to be used in surveying other sites or to be directed to management. Hence, “removal” sampling will ultimately result in a more efficient invasive management program, as we have demonstrated. In some situations, however, the sampling will necessarily be of the “nonremoval” type. For instance, the presence of invasive species might be monitored through the use of camera traps, which are left recording for a given amount of time with detections only being identified after camera recollection. Other examples include surveys that require the postprocessing of collected samples, for example, when species identification is carried out via genetic analysis.

Regardless of whether “removal” or “nonremoval” sampling is used, the optimization results indicate that more intensive surveillance should be applied to sites that are more likely to contain the species and where the savings due to early detection are large. It is also under these conditions that the difference in efficiency between the two sampling methods is greater. Compared to the “nonremoval” case, the optimal surveillance solution for a program based on “removal” sampling initially requests more sampling effort to be applied to all sites. However, this effort only needs to be fully spent in those sites where the species is not detected. We can interpret this as follows: under the “removal” design, the optimization requests more sampling effort to establish species absence at a site, and this effort is “borrowed” from sites where the species is detected. By increasing the chances of detecting the species, the program obtains more savings from its early management.

It is important to note that the cost optimization methodology used is based on expectations, which are a function of the estimated values for the occupancy probabilities and detection rates. There are some implications to this. For instance, let us consider the case where survey budget is constrained. While the optimal solution for the “removal” sampling surveillance ensures that the *expected* survey costs will be within budget, in a practical implementation, the actual survey costs might be somewhat larger or smaller due to stochasticity. While this can be easily dealt with by leaving some moderate room in the budget for contingency costs, a bigger problem is where the parameter values used in the optimization do not provide a reasonably good representation of the true occupancy probabilities or detection rates characterizing the system. In such case, the actual survey budget spent could be considerably larger than expected. Hence, it is recommended to monitor the progress of the surveillance program, adapting the strategy if notable disparities are identified between the assumed and apparent occupancy probabilities and detection rates. This is relevant not only when the budget for surveys is tightly constrained as, in any case, the misspecification of the parameter values will force the surveillance strategy away from the optimal solution. In a “nonremoval” design, the amount of effort actually spent in surveying the sites is ensured to be within the allowed budget, as surveys costs are not dependent on whether the species is detected or not. However, this strategy will also suffer from misspecification of the parameter values and an implementation involving potential adaptation will also be required for the good performance of the program (Hauser and McCarthy 2009). Alternatively, the surveillance design could explicitly incorporate parameter uncertainty (McCarthy et al. 2010).

Hauser and McCarthy (2009) discuss the sensitivity of the optimal surveillance solution with respect to variation in the occupancy estimates. In particular, they found that, for a “nonremoval” design with a constrained surveillance budget, it is only the relative probabilities of occurrence and the benefits of detection that determine the optimal allocation. However, this does not hold when the survey budget is unconstrained (or when it is constrained but remains effectively unconstrained, i.e., *B* > B*), as in this case, the optimal solution is a function of the absolute values of the occupancy probabilities. We have shown that, under the “removal” sampling, the absolute values of the probabilities of occupancy also matter, and this applies regardless of whether the survey budget is or not constrained. As an example of the impact of a scaling in the occupancy probabilities, consider again the scenario in Fig. 5A. If the optimal allocation of effort were determined based on the scaled probabilities, with the true ones being the original ones, then the total costs would increase by 14% in the unconstrained case. A scaling by 0.3 would result in a 69% total cost increase. This sensitivity of the optimal surveillance solution to the scaling in occupancy probabilities calls for caution with respect to the estimates fed into the optimization algorithm. For instance, it is worth noting that species distribution modeling methods based on presence-only records can only estimate relative likelihood of species presence (Elith et al. 2011; Fithian and Hastie 2013). Unless there are some means for recalibrating their output, using the estimates from such models will produce a suboptimal solution in the allocation problem.

Managing invasive species is increasingly important, both in terms of biodiversity conservation and protecting economic systems. Monitoring and management must be combined when dealing with invasive species. Addressing the trade-off between these two activities leads to more efficient invasive management programs, ultimately leading to reduced overall costs. We show how the prevalence of the species as well as its detectability and the benefits derived from early detection should be considered. Stopping surveys after detection increases efficiency, with the gains being fully realized when this is considered when designing a surveillance program.

## Supporting Information

**Appendix S1.** Derivation of the expected survey length expression.

**Appendix S2.** Optimal survey allocation with no budgetary constraint (site-by-site) for a “removal” design.

**Appendix S3.** Optimal survey allocation with constrained survey budget (multisite) for a “removal” design.

**Appendix S4.** Comparison of requested survey length between the “removal” and “nonremoval” design (unconstrained scenario).

**Appendix S5.** Additional figure.

**Appendix S6.** Computer code.
