# Supplementary material for: Optimal surveillance strategy for invasive species management when surveys stop after detection
Source: Ecol Evol. 2014 Apr 11;4(10):1751–60. doi: 10.1002/ece3.1056 (PMC4063473; doi:10.1002/ece3.1056)
Supplement: Supplementary file 1 [file ece30004-1751-SD1.pdf]

S0 – Notation

γ_i: detection rate at site i

ψ_i: occupancy probability at site i

L_i: (actual) survey length at site i

L_{M_i}: maximum survey length at site i (design requirement)

γ_i^*: probability of detecting the species (at least once) at site i in a survey length L_{M_i}

C_{D_i}: cost of early management at site i (in survey cost units)

C_{U_i}: cost of late management at site i (in survey cost units)

C_{T_i}: total costs (management and survey) at site i (in survey cost units)

B: survey budget (allowed overall survey cost across all sites)

B^*: optimal survey budget for unconstrained case

S1 – Derivation of the expected survey length expression

Let us assume that the survey at a site only lasts until the species is detected, up to a maximum duration of L_{M_i}. The expected length of a survey at an occupied site i is computed as

$$\mathbb{E}[L_i|\text{occ}] = \int_0^{L_{M_i}} l f_l(l)\, dl\,,$$

where $f_l(l)$ is the probability density function of the survey lengths. The form of $f_l(l)$ is that of an exponential distribution, but truncated to a maximum length L_{M_i}, that is

$$f_l(l) = \begin{cases} \gamma_i e^{-\gamma_i l}, & l < L_{M_i} \\ e^{-\gamma_i L_{M_i}}, & l = L_{M_i} \end{cases},$$

where $e^{-\gamma_i L_{M_i}}$ is the probability of having no detections in a survey of length L_{M_i}. Hence, we have that

$$\mathbb{E}[L_i|\text{occ}] = \gamma_i \int_0^{L_{M_i}} l e^{-\gamma_i l} dl + L_{M_i} e^{-\gamma_i L_{M_i}}\,.$$

Taking into account that $\int xe^{cx}dx = e^{cx}(cx-1)/c^2$, and denoting $\gamma_i^* = 1 - e^{-\gamma_i L_{M_i}}$, we have that

$$\mathbb{E}[L_i|\text{occ}] = \frac{-e^{-\gamma_i L_{M_i}}\left(\gamma_i L_{M_i}+1\right)}{\gamma_i} + \frac{1}{\gamma_i} + L_{M_i}e^{-\gamma_i L_{M_i}} = \frac{1-e^{-\gamma_i L_{M_i}}}{\gamma_i} = \frac{\gamma_i^*}{\gamma_i}.$$

S2 – Optimal survey allocation with no budgetary constraint (site-by-site) when surveys stop after detection ('removal' design)

We find the optimal amount of (maximum) survey effort as that which minimizes the expected total cost at site i

$$\begin{aligned}\mathbb{E}\left[C_{T_i}\right] &= C_{S_i}\mathbb{E}[L_i] + C_{D_i}\Pr(\text{occ}\&det) + C_{U_i}\Pr(\text{undet}\&occ)\\ &= C_{S_i}\mathbb{E}[L_i] + C_{D_i}\psi_i\gamma_i^* + C_{U_i}\psi_i(1-\gamma_i^*)\\ &= C_{S_i}\mathbb{E}[L_i] + C_{D_i}\psi_i + \left(C_{U_i}-C_{D_i}\right)\psi_i(1-\gamma_i^*)\,.\end{aligned}$$

Without loss of generality we hereafter assume that costs are expressed in terms of units of survey cost, so that $C_{S_i} = 1$, and we denote $C_{U_i} - C_{D_i} = \Delta C_i$

$$\mathbb{E}\left[C_{T_i}\right] = \mathbb{E}[L_i] + C_D\psi_i + \Delta C_i\psi_i(1-\gamma_i^*)\,. \quad \text{(S2.1)}$$

Considering a sampling protocol in which surveys stop after detection we have that the expected survey costs are (Appendix S1)

$$\mathbb{E}[L_i] = (1-\psi_i)L_{M_i} + \psi_i\,\gamma_i^*/\gamma_i.$$

Differentiating (S2.1) with respect to L_{M_i} and equating to zero we have that

$$\left[(1-\psi_i) + \frac{\psi_i}{\gamma_i}\frac{d\gamma_i^*}{dL_{M_i}}\right] - \Delta C_i\psi_i\frac{d\gamma_i^*}{dL_{M_i}} = 0,$$

where $d\gamma^*/dL_{M_i} = \gamma_i e^{-\gamma_i L_{M_i}}$, that is,

$$(1-\psi_i) + (1-\Delta C_i\gamma_i)\psi_i e^{-\gamma_i L_{M_i}} = 0, \quad \text{(S2.2)}$$

which leads to

$$e^{-\gamma_i L_{M_i}} = \frac{1-\psi_i}{\psi_i}\frac{1}{\gamma_i \Delta C_i - 1},$$

and

$$L_{M_i} = \frac{1}{\gamma_i}\ln\left[\frac{\psi_i}{1-\psi_i}(\gamma_i \Delta C_i - 1)\right].$$

To ensure that L_{M_i} is larger than zero we need

$$\frac{\psi_i}{1-\psi_i}(\gamma_i \Delta C_i - 1) > 1,$$

which leads to

$$\Delta C_i \psi_i \gamma_i > 1.$$

Therefore we have that

$$L_{M_i} = \begin{cases} \frac{1}{\gamma_i}\ln\left[\frac{\psi_i}{1-\psi_i}(\gamma_i \Delta C_i - 1)\right], & \text{if } \Delta C_i \psi_i \gamma_i > 1 \\ 0, & \text{otherwise.} \end{cases} \quad \text{(S2.3)}$$

S3 – Optimal survey allocation with constrained survey budget (multi-site) when surveys stop after detection

We now consider the problem in S2 for the case where the total survey budget is constrained. We solve this using the Kuhn-Tucker conditions, which state that the critical point must satisfy

$$\frac{\partial f}{\partial x_i} + \sum_{j=1}^{n} \lambda_j \frac{\partial g_j}{\partial x_i} + \lambda_{n+1} \frac{\partial g_{n+1}}{\partial x_i} = 0, \;\; i = 1 \dots n \qquad \text{(KT1)}$$

$$\lambda_i (b_i - g_i) = 0, \;\; i = 1 \dots n+1 \qquad \text{(KT2)}$$

$$\lambda_i \geq 0, \;\; i = 1 \dots n+1 \qquad \text{(KT3)}$$

where f is the objective function and g are the constraints.

→ STEP 1a (formulation): The aim is to minimize the overall total costs subject to the constraint, so the problem formulation is as follows

$$\min f(\boldsymbol{L_M}) = \textstyle\sum_{i=1}^{n} \mathbb{E}\left[C_{T_i}\right] = \sum_{i=1}^{n} \left\{\mathbb{E}[L_i] + C_{D_i}\psi_i + \Delta C_i \psi_i \exp\left(-\gamma_i L_{M_i}\right)\right\}$$

subject to

$$g_i(\boldsymbol{L_M}) = -L_{M_i} \leq 0, \;\; i = 1 \dots n$$

$$g_{n+1}(\boldsymbol{L_M}) = \sum_{i=1}^{n} \mathbb{E}[L_i] \leq B,$$

where $\mathbb{E}[L_i] = (1-\psi_i)L_{M_i} + \psi_i \left[1 - \exp(-\gamma_i L_{M_i})\right] / \gamma_i$.

→ STEP 2a: From (KT1) we have

$$\frac{\partial \mathbb{E}[L_i]}{\partial L_{M_i}} + \Delta C_i \psi_i \exp\left(-\gamma_i L_{M_i}\right)(-\gamma_i) - \lambda_i + \lambda_{n+1} \frac{\partial \mathbb{E}[L_i]}{\partial L_{M_i}} = 0, \;\; i = 1 \dots n \qquad \text{(S3.1)}$$

where

$$\frac{\partial \mathbb{E}[L_i]}{\partial L_{M_i}} = (1-\psi_i) + \psi_i \exp\left(-\gamma_i L_{M_i}\right). \qquad \text{(S3.2)}$$

→ STEP 3a (ineq. B): From (KT2) we have that either $\lambda_{n+1} = 0$ or $\sum_{i=1}^{n} \mathbb{E}[L_i] = B$. It is obvious that, if the total allowed survey budget is larger than the budget required in the unconstrained problem in section S2 ($B > B^*$), then the optimal solution is that in section S2 and not all the survey budget is to be used ($\lambda_{n+1} = 0$ and $\sum_{i=1}^{n} \mathbb{E}[L_i] < B$). If, on the other hand, the survey budget is smaller than then budget required in the unconstrained optimal design ($B < B^*$), then the optimal solution involves using all the available survey effort as we show here below.

If $\lambda_{n+1} = 0$ from (S3.1) we have

$$(1-\psi_i) + \psi_i \exp(-\gamma_i L_{M_i}) - \Delta C_i \psi_i \gamma_i \exp(-\gamma_i L_{M_i}) - \lambda_i = 0, \;\; i = 1 \dots n \qquad \text{(S3.3)}$$

From (KT2) we have that for each $i = 1 \dots n$ either $\lambda_i = 0$ or $L_{M_i} = 0$. Let S be the set of sites for which $L_{M_i} > 0$ (and $\lambda_i = 0$), and S' the set of sites with $L_{M_i} = 0$ (and $\lambda_i \geq 0$). For sites in S we have

$$(1-\psi_i) + \psi_i \exp(-\gamma_i L_{M_i}) - \Delta C_i \psi_i \gamma_i \exp(-\gamma_i L_{M_i}) = 0, \;\; i = 1 \dots n$$

which is the same as (S2.2) and essentially leads to the optimal unconstrained solution in (S2.3). However this solution is not possible when $B < B^*$, as there is no budget to fulfill it. This implies that when $B < B^*$, $\lambda_{n+1} \neq 0$ and hence $\sum_{i=1}^{n} \mathbb{E}[L_i] = B$.

→ STEP 1' (re-formulation): for simplicity we can reformulate the problem removing from the objective function the parts that are independent of $\boldsymbol{L_M}$. Considering that $B < B^*$, the problem is equivalent to

$$\min f(\boldsymbol{L_M}) = \textstyle\sum_{i=1}^{n} \Delta C_i \psi_i \exp(-\gamma_i L_{M_i}) \;\; \text{subject to}$$

$$g_i(\boldsymbol{L_M}) = -L_{M_i} \leq 0, \;\; i = 1 \dots n$$

$$g_{n+1}(\boldsymbol{L_M}) = \sum_{i=1}^{n} \mathbb{E}[L_i] \leq B,$$

→ STEP 2': From (KT1) we have

$$\Delta C_i \psi_i \exp(-\gamma_i L_{M_i})(-\gamma_i) - \lambda_i + \lambda_{n+1} \frac{\partial \mathbb{E}[L_i]}{\partial L_{M_i}} = 0, \;\; i = 1 \dots n. \qquad \text{(S3.4)}$$

→ STEP 3': As shown above, we have that $\lambda_{n+1} > 0$ and $\sum_{i=1}^{n} \mathbb{E}[L_i] = B$.

→ STEP 4 (get L_{M_i} and λ_i): For all sites in S ($L_{M_i} > 0, \lambda_i = 0$) we get from (S3.4)

$$\lambda_{n+1}(1-\psi_i) + \exp(-\gamma_i L_{M_i})\,\psi_i(\lambda_{n+1} - \Delta C_i\,\gamma_i) = 0, \quad i = 1 \ldots n$$

and from this we have that

$$\exp(-\gamma_i L_{M_i}) = \frac{1-\psi_i}{\psi_i}\frac{\lambda_{n+1}}{\Delta C_i\,\gamma_i - \lambda_{n+1}}, \qquad \text{(S3.5)}$$

$$L_{M_i} = \frac{1}{\gamma_i}\ln\left(\frac{\psi_i}{1-\psi_i}\frac{\Delta C_i\,\gamma_i - \lambda_{n+1}}{\lambda_{n+1}}\right). \quad \text{(S3.6)}$$

For all sites in S' ($L_{M_i} = 0, \lambda_i \geq 0$) we have from (S3.4) that

$$\lambda_i = \lambda_{n+1} - \Delta C_i \psi_i \gamma_i. \quad \text{(S3.7)}$$

→ STEP 5 (get λ_{n+1}): Substituting in $\sum_{i=1}^{n} \mathbb{E}[L_i] = B$ we get that λ_{n+1} must fulfill

$$\sum_{i=1}^{n} \mathbb{E}[L_i] = \sum_{i\in S}\left\{(1-\psi_i)L_{M_i} + \frac{\psi_i}{\gamma_i}\left[1 - \exp(-\gamma_i L_{M_i})\right]\right\}$$

$$= \sum_{i\in S}\left\{\frac{1-\psi_i}{\gamma_i}\ln\left(\frac{\psi_i}{1-\psi_i}\frac{\Delta C_i\,\gamma_i - \lambda_{n+1}}{\lambda_{n+1}}\right) + \frac{1}{\gamma_i}\frac{\psi_i \Delta C_i \gamma_i - \lambda_{n+1}}{\Delta C_i \gamma_i - \lambda_{n+1}}\right\} = B, \text{(S3.8)}$$

and can be obtained numerically.

→ STEP 6 (conditions): To ensure that $L_{M_i} > 0$ for $i \in S$ we need

$$\frac{\psi_i}{1-\psi_i}\frac{\Delta C_i\,\gamma_i - \lambda_{n+1}}{\lambda_{n+1}} > 1,$$

that is $\lambda_{n+1} < \Delta C_i \psi_i \gamma_i$. For $i \in S'$ we have that $L_{M_i} = 0$ and $\lambda_i \geq 0$ so, given (S3.7), we have that $\Delta C_i \psi_i \gamma_i \leq \lambda_{n+1}$. Therefore we can prioritize our sites in terms of $\Delta C_i \psi_i \gamma_i$ in descending order. Provided now that the sites are arranged in this way, the conditions above are summarized by

$$\Delta C_s \psi_s \gamma_s > \lambda_{n+1} \geq \Delta C_{s+1} \psi_{s+1} \gamma_{s+1},$$

where s is the number of sites in the set S.

S4 – Comparison of requested survey length between the 'removal' and 'non-removal' design (unconstrained scenario)

The optimal survey length is for a 'removal' design

$$L_{M_i} = \begin{cases} \frac{1}{\gamma_i} \ln\left[\frac{\psi_i}{1-\psi_i}(\gamma_i \Delta C_i - 1)\right], & \text{if } \gamma_i \psi_i > \frac{1}{\Delta C_i} \\ 0, & \text{otherwise,} \end{cases}$$

and for a 'non-removal' design

$$L_{M_i} = \begin{cases} \frac{1}{\gamma_i} \ln(\psi_i \gamma_i \Delta C_i), & \text{if } \gamma_i \psi_i > \frac{1}{\Delta C_i} \\ 0, & \text{otherwise.} \end{cases}$$

If under the 'removal' design the optimal survey length were larger than under a 'non-removal' design we would have

$$\frac{\psi_i}{1-\psi_i}(\gamma_i \Delta C_i - 1) > \psi_i \gamma_i \Delta C_i.$$

By manipulating this inequality through basic algebra we arrive to

$$\gamma_i \psi_i > \frac{1}{\Delta C_i},$$

hence, whenever $L_{M_i} > 0$, L_{M_i} is indeed larger under the 'removal' design.

S5 – Additional figure

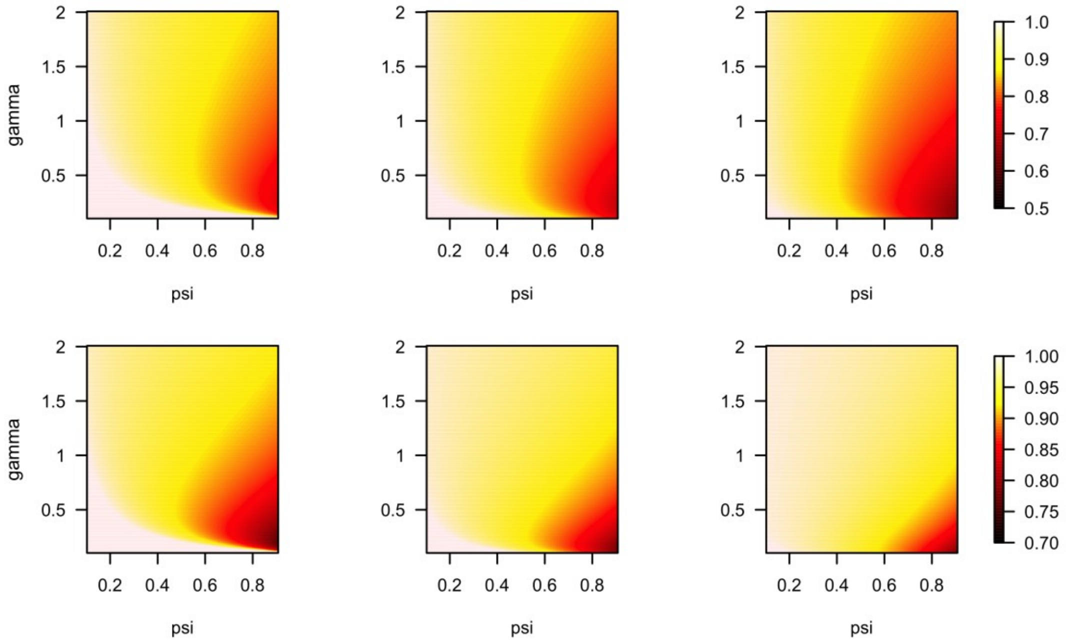

Figure S5.1: As Figure 2 in main text but with $C_D = 10$.
